# Assessment of the Impacts of *Centipeda minima* (L.) on Cell Viability, and Osteogenic Differentiation of Mesenchymal Stem Cell Spheroids

**DOI:** 10.3390/medicina59010043

**Published:** 2022-12-26

**Authors:** Hyun-Jin Lee, Kyung-Hwan Na, Md. Salah Uddin, Jun-Beom Park

**Affiliations:** 1 Department of Periodontics, College of Medicine, the Catholic University of Korea, Seoul 06591, Republic of Korea; 2 Department of Medicine, Graduate School, the Catholic University of Korea, Seoul 06591, Republic of Korea; 3 Ethnobotanical Database of Bangladesh, Tejgaon, Dhaka 1208, Bangladesh

**Keywords:** Asteraceae, bone marrow, cell differentiation, cellular spheroids, cell survival, herbal medicine, stem cells

## Abstract

*Background and Objectives: Centipeda minima* (L.) is a well-known and traditional pharmaceutical that has been utilized to treat different conditions controlling rhinitis, soothe pain, and decrease swelling. We assessed the impacts of *Centipeda minima* (L.) extricates (CMTs) on the osteogenic differentiation of cell spheroids made of human-bone-marrow-derived mesenchymal stem cells. *Materials and Methods:* Mesenchymal stem cells (MSCs) in spheroid 3D culture were generated and propagated in the presence of CMTs ranging from 0 to 1 μg/mL. Cell morphology was measured on Days 1, 3, 5, and 7. The quantitative cellular viability was evaluated on Days 1, 3, 5, and 7. Alkaline phosphatase activity assays were designed to measure the osteogenic differentiation of mesenchymal stem cell spheroids on Day 7. Alizarin Red S staining was performed to investigate the mineralization of cell spheroids on Days 7 and 14. Real-time polymerase chain reactions were used to measure the expression levels of RUNX2 and COL1A1 on Day 14. Western blot techniques were performed to identify the protein expression of Runt-related transcription factor 2 and type I collagen. *Results:* The control group’s mesenchymal stem cells displayed a spheroid shape. There was no noticeable change in morphology with the addition of CMTs at final concentrations of 0.001, 0.01, 0.1, and 1 μg/mL compared with the untreated (control) group. The application of CMTs did not induce a significant change in cell viability. The relative alkaline phosphatase activity values in the 0.001, 0.01, 0.1, and 1 μg/mL CMT groups were 114.4% ± 8.2%, 130.6% ± 25.3%, 87.8% ± 3.4%, and 92.1% ± 6.8%, respectively, considering a control of 100% (100.0% ± 17.9%). On Day 14, calcium deposits were clearly observed in each group. The relative values of Alizarin Red S staining in the 0.001, 0.01, 0.1, and 1 μg/mL CMT groups were 100.1% ± 8.9%, 105.9% ± 0.0%, 109.7% ± 19.1%, and 87.0% ± 40.9%, respectively, considering a control of 100% (100.0% ± 28.7%). The addition of CMT significantly increased RUNX2 expression in the 0.01 μg/mL group and COL1A1 in the 0.001 and 0.01 μg/mL groups. Normalization of protein expression showed that the addition of CMTs significantly increased type I collagen expression in the 0.001, 0.01, and 1 μg/mL groups. *Conclusions:* In conclusion, CMTs influence the osteogenic differentiation of bone-marrow-derived mesenchymal stem cells and the use of CMTs may positively influence the osteogenic differentiation of cell spheroids.

## 1. Introduction

*Centipeda minima* (L.) A. Br. et Aschers has long been used in Oriental medicine for swelling, detoxification, and clearing orifices [1]. *Centipeda minima* (L.), a traditional Chinese herbal medicine used to relieve pain and reduce swelling, has recently been shown to be overwhelmingly effective with varying response rates against breast, colon, and nasopharyngeal cancer, exerting antitumor effects [2]. It has been shown to possess anti-inflammatory and antioxidant properties [3]. Moreover, *Centipeda minima* (L.) essential oil is a multi-target agent and provides a new research platform and represents a reference for the treatment of allergic rhinitis [4].

Mesenchymal-stem-cell-based therapy reportedly has potential for bone repair [5]. In several drug delivery research papers, 2D and 3D cultures were compared and presented; thus, the differences and effects in 2D were insignificant, but these difference in results in 3D were noticeable and proved to be useful materials for many applications [6,7]. Three-dimensional (3D) cell aggregates can imitate the natural microenvironment [8]. Small adipose-derived stem cell spheroids cultured in scaffold-free three-dimensional condition survived and promoted bone regeneration under in vitro and in vivo conditions [9]. Small injectable adipose stem cell spheroids are suggested to be a less invasive treatment alternative for the treatment of bony defects [9]. The three-dimensional spheroid culture of stem cells increased the production of key inflammatory regulators [10]. *Centipeda minima* could be a major effector of the Wnt/β-catenin signaling pathway. Wnt signaling to target genes through beta-catenin increases the expression of known cyclins D1 and c-myc. Such Wnt/beta-catenin signaling is reported to not only be involved in stem cell proliferation, but also in some stem cell differentiation processes [11,12,13]. This study was performed to analyze the effect of *Centipeda minima* (L.) extract (CMT) on preserving morphology, developing cell viability, and promoting the osteogenic differentiation of mesenchymal stem cell spheroids.

## 2. Materials and Methods

### 2.1. Preparation of Plant Materials

*Centipeda minima* (L.) was collected from Sonaimuri sub-district, Noakhali district, Chittagong division in Bangladesh by Md. Salah Uddin. The specimen recorded as KRIB 0054834 has been deposited at the herbarium of the Korea Research Institute of Bioscience and Biotechnology. After drying and grinding the whole plant of *Centipeda minima* (L.), 70 g of powder was extracted with 1 L of 99.9% (*v*/*v*) methanol, repeatedly sonicated (15 min), and left undisturbed at 45 °C for 3 days. The resulting product was filtered through non-fluorescent cotton, concentrated under reduced pressure on a rotary evaporator (N-1000SWD, EYELA, Tokyo, Japan) at 45 °C, and lyophilized to obtain 5.23 g of CMT. The final product was 24.70 mg ± 0.17 mg.

### 2.2. The Method Used for Producing Spheroid

StemFIT 3D (MicroFIT Co., Ltd., Gyeonggi-do, Republic of Korea) was used to form uniformly sized spheroids. The experiment was carried out in the following order. Sterilized and vacuumed packages were removed. StemFIT 3D was placed in a Petri dish and 70% ethanol was added. Bubbles inside the well formed when ethanol was added were removed by pipetting. Ethanol inside the wells was removed and StemFIT 3D was filled with the culture medium to replace ethanol inside the wells. Single cell suspension was evenly applied to the center of the StemFIT 3D. Depending on the type of cell, spheroids formed over 5 to 24 h.

### 2.3. Study Design Using Bone-Marrow-Derived Mesenchymal Stem Cells

**Figure 1:**
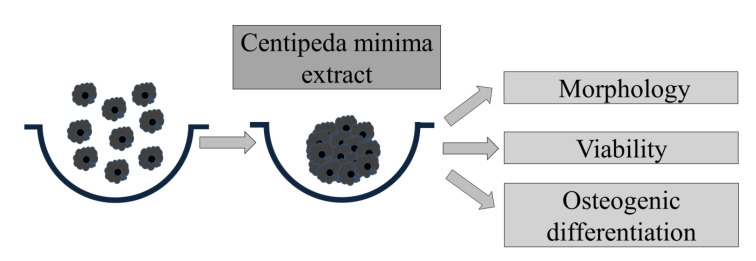
An illustration of the design of the current study.

The study protocol was reviewed and approved by the Institutional Review Board of Seoul St. Mary’s Hospital (approval numbers KC20SISE0839 and KC22SISE0030; approval date, 3 November 2020). Informed consent was acquired from the female participant. All tests were conducted in conformity with the pertinent rules and regulations outlined in the Helsinki Declaration. Figure 1 presents an overview of the study design. The Catholic Institute of Cell Therapy provided mesenchymal stem cells produced from the human bone marrow of a male participant (Catholic MASTER Cells) (CIC, Seoul, Republic of Korea) [14]. Media were changed every one to two days. Cells were grown in an incubator at 37 °C, 95% O_2_, and 5% CO_2_.

### 2.4. Evaluation of Cell Morphology

Mesenchymal stem cells were seeded at a density of 1.0 × 10^6^ cells/well and cultured in osteogenic medium (alpha-minimal essential medium (α-MEM, Gibco, Grand Island, NY, USA) supplemented with fetal bovine serum (FBS, Gibco, Grand Island, NY, USA), dexamethasone, ascorbic acid 2-phosphate (Sigma-Aldrich Co., St. Louis, MO, USA), glycerophosphate disodium salt hydrate, L-glutamine (Sigma-Aldrich Co.), 100 U/mL penicillin, and 100 μg/mL streptomycin (Sigma-Aldrich Co.) [15]. Final concentrations of CMTs were 0, 0.001, 0.01, 0.1, and 1 μg/mL, respectively. Morphological evaluations were performed using an inverted microscope on Days 1, 3, 5, and 7 (CKX41SF, Olympus Corporation, Tokyo, Japan). Spheroid stem cell diameters were measured by analyzing reference lengths on Days 1, 3, 5, and 7 [16].

### 2.5. Assessment of Cell Viability 

Using the Counting Kit-8, the assessment of cellular viability was carried out on Days 1, 3, 5, and 7 in accordance with the prior report (CCK-8, Dojindo, Tokyo, Japan) [17]. Cells were exposed to tetrazolium monosodium salt for one hour at room temperature. Utilizing a microplate reader, spectrophotometric absorbance at 450 nm was measured (BioTek Instruments Inc., Winooski, VT, USA).

### 2.6. Tests for Alkaline Phosphatase Activity

Using a commercially available kit, alkaline phosphatase activity assays were performed on Day 7 of the experiment (K412-500, BioVision, Inc., Milpitas, CA, USA). To gauge the samples’ spectrophotometric absorbance, a microplate reader was employed [18].

### 2.7. Alizarin Red S Staining Evaluation

Alizarin Red S staining was conducted on Days 7 and 14. Washing, fixing, and staining with 2% Alizarin Red S Solution (ScienCell Research Laboratories, Inc., Carlsbad, CA, USA) were all performed on the cells and scrutinized under a microscope (CKX41SF, Olympus Corporation) [19]. Using image analysis software to measure the staining’s intensity, the relative values of Alizarin Red S staining were calculated (ImageJ, National Institutes of Health, Bethesda, MD, USA) [20].

### 2.8. Real-Time Quantitative Polymerase Chain Reaction for the Measurement of RUNX2 and COL1A1 mRNA 

A commercially available kit was used to perform total RNA extraction (Thermo Fisher Scientific, Inc., Waltham, MA, USA), following the manufacturer’s specifications [21]. We used the kit (RNA 6000 Nano Chip; Agilent Technologies, Santa Clara, CA, USA) to assess the quality of RNA, and a spectrophotometer was used to assess the quantity of RNA based on the ratio of absorbance at 260 nm and 280 nm (ND-2000, Thermo Fisher Scientific, Inc., Waltham, MA, USA). Reverse transcriptase (SuperScript II; Invitrogen, Carlsbad, CA, USA) was utilized with RNA as a template for reverse transcription.

Using real-time quantitative polymerase chain reaction (qPCR), gene expression was measured and analyzed to accurately assess cell differentiation kinetics. The sense and antisense PCR primers were created using GenBank. The primer sequences used are as follows: RUNX2 (accession number: NM_001015051.3; forward: 5′-CAGTTCCCAAGCATTTCATCC-3′, reverse: 5′-AGGTGGCTGGATAGTGCATT-3′), COL1A1 (accession number: NM_000088.4; forward: 5′-TACCCCACTCAGCCCAGTGT-3′, reverse: 5′-CCGAACCAGACATGCCTCTT-3′), and β-actin (accession number: NM 001101: forward: 5′-AATGCTTCTAGGCGGACTATGA-3′, reverse: 5′-TTTCTGCGCAAGTTAGGTTTT-3′). The β-actin housekeeping gene was used for normalization. Using the readily available commercial kit (TOPreal™ qPCR 2X PreMIX, Enzynomics, Daejeon, Republic of Korea), real-time PCR was carried out using the PCR System (StepOnePlus^TM^; Applied Biosystems; Thermo Fisher Scientific, Inc. Waltham, MA, USA), observing the directions provided by the manufacturer [16,22].

### 2.9. Runt-Related Transcription Factor 2 and Type I Collagen Western Blot Analysis

Samples were processed with a lysis buffer for 30 min after being washed twice with ice-cold PBS. At 4 °C, the lysates were centrifuged for 15 min at 12,000× *g*. Gel separation (Mini-PROTEAN^®^ TGX™ Precast Gels; Bio-Rad, Hercules, CA, USA), transblotting to membranes (Immun-Blot^®^; Bio-Rad), and immunoblotting with the appropriate antibodies and detection kits were all used to separate the samples.

Primary antibodies against Runt-related transcription factor 2 (RUNX2, ab76956; Abcam, MA, USA), collagen I (ab6308; Abcam), and glyceraldehyde 3-phosphate dehydrogenase (GAPDH, ab9485; Abcam) were used for the analysis and secondary antibodies were purchased from Abcam. Using an image processing tool (ImageJ, National Institutes of Health, Bethesda, MD, USA), the protein expressions of RUNX2, collagen I, and GAPDH were quantitatively evaluated.

### 2.10. Statistical Evaluation

The results of the experiments are displayed as means and standard deviations. Using a commercially available program (SPSS 12 for Windows, SPSS Inc., Chicago, IL, USA), tests for normality and a one-way analysis of variance with post hoc Tukey’s test were carried out to ascertain the differences between the groups. The significance threshold was set at 0.05.

## 3. Results

### 3.1. Analyzing the Morphology of the Mesenchymal Stem Cell Spheroids

**Figure 2:**
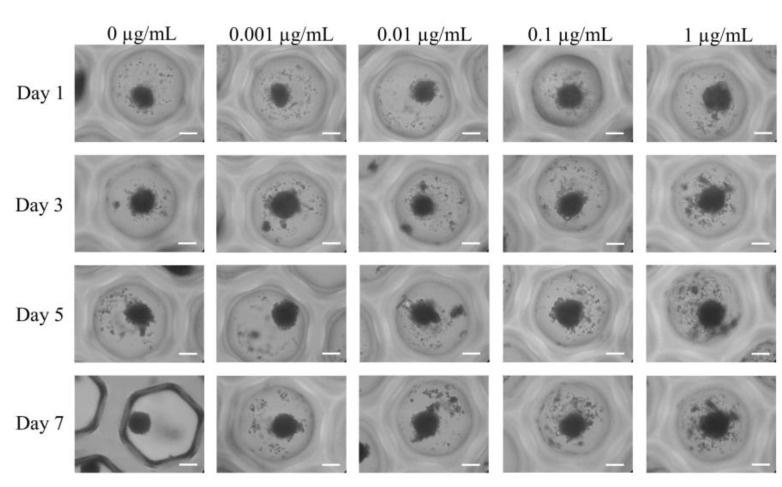
The morphology of the cell spheroids on Days 1, 3, 5, and 7 after exposure to various CMT concentrations in osteogenic medium was assessed using inverted microscopy (original magnification × 200). The bar shows 200 μm.

Figure 2 displays the shape of mesenchymal stem cell spheroids exposed to CMT on Day 1 at final concentrations of 0, 0.001, 0.01, 0.1, and 1 μg/mL. On Day 1, the control group’s mesenchymal stem cell spheroids displayed a round shape. When compared with the untreated control group, the morphology of mesenchymal stem cell spheroids in the presence of CMT at final doses of 0.001, 0.01, 0.1, and 1 μg/mL did not exhibit any discernible modifications. After Days 3, 5, and 7 of extended incubation, no morphological alterations were exhibited.

**Figure 3:**
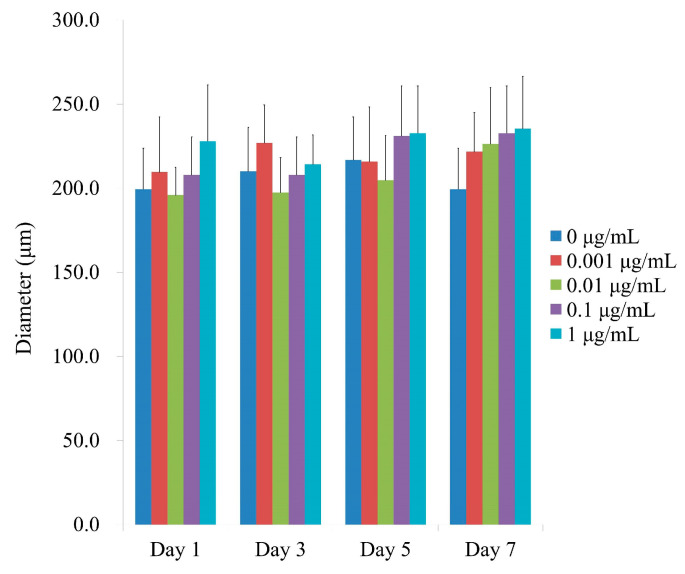
Spheroid diameters on Days 1, 3, 5, and 7. On Day 1, CMT application did not reveal any statistically significant differences (*p >* 0.05). Additionally, no statistically significant alterations were detected between the groups in the longer culture period (*p >* 0.05).

Figure 3 shows the average spheroid sizes on Days 1, 3, 5, and 7 when CMT was present at final concentrations of 0, 0.001, 0.01, 0.1, and 1 μg/mL. No statistically significant differences were found between groups on Day 1 (*p >* 0.05). In addition, no statistically significant changes were found between the groups that received CMT and the control group with a lengthier incubation period (*p >* 0.05).

### 3.2. Calculation of the Quantitative Viability of Spheroids

**Figure 4:**
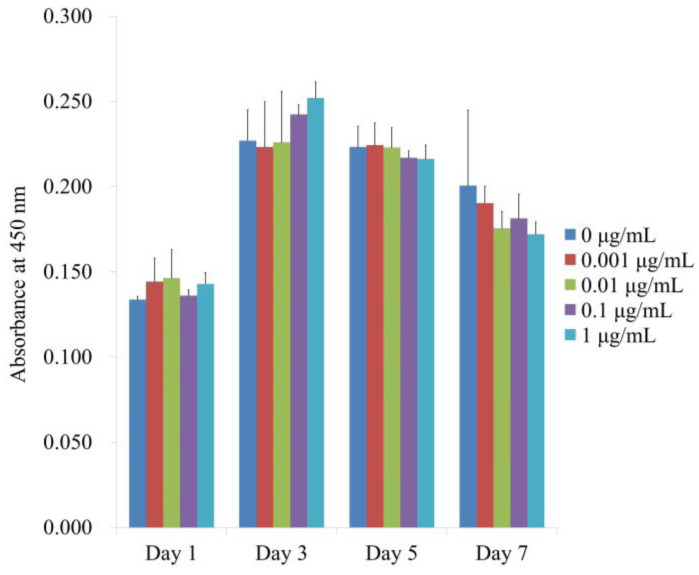
Days 1, 3, 5, and 7 of the CCK-8 assay for cellular viability. Cellular viability was not significantly altered by the application of CMT (*p >* 0.05). Additionally, there were no discernible differences between the groups with prolonged incubation durations (*p >* 0.05).

Figure 4 displays the numerical outcomes for cellular viability on Days 1, 3, 5, and 7. When the control was taken to be 100% (100.0% ± 1.6%), the relative values for CMT at concentrations of 0.001, 0.01, 0.1, and 1 μg/mL were 108.0% ± 10.3%, 109.5% ± 12.7%, 101.7% ± 2.6%, and 107.0% ± 4.9%, respectively. Cellular viability was not significantly altered by the application of CMT (*p >* 0.05). Additionally, no discernible variations between the groups were found with longer incubation periods (*p >* 0.05).

### 3.3. Tests for Alkaline Phosphatase Activity

**Figure 5:**
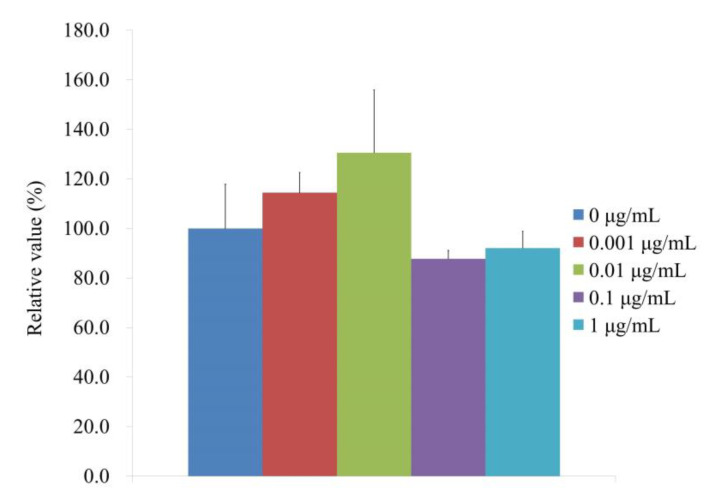
Activity of alkaline phosphatase on Day 7.

Figure 5 displays the ALP and CCK-8 activity at Day 7, which indicates the alkaline phosphatase activity treated with CMT on Day 7. When the control was taken to be 100% (100.0% ± 17.9%), the relative alkaline phosphatase activity values for the 0.001, 0.01, 0.1, and 1 μg/mL CMT groups were 114.4% ± 8.2%, 130.6% ± 25.3%, 87.8%± 3.4%, and 92.1% ± 6.8%, respectively. The findings showed that, on Day 7, there were there were no statistically significant changes when compared with the unloaded group (*p >* 0.05).

### 3.4. Assay for Mineralization

**Figure 6:**
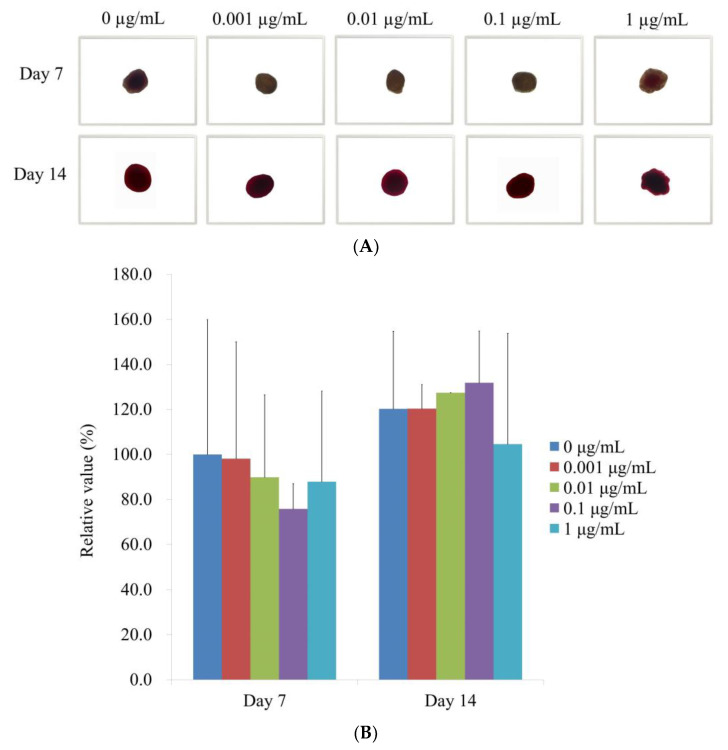
Alizarin Red S staining is used to assess mineralization. (**A**) Analysis of Alizarin Red S staining at the microscopic level on Days 7 and 14 (original magnification × 200). The scale bar indicates 100 μm. (**B**) Quantitative evaluation of Days 7 and 14 Alizarin Red S staining.

Figure 6A displays the results of the Alizarin Red S staining on Days 7 and 14 after providing with various concentrations of CMT in an osteogenic supplement. On Day 14, it was clearly seen calcium deposits in each group. When control was taken as 100% (100.0% ± 59.9%), the relative values of Alizarin Red S staining on Day 7 for the 0.001, 0.01, 0.1, and 1 g/mL CMT groups were, 98.1% ± 51.9%, 89.8% ± 36.6%, 75.8% ± 11.2%, and 87.9% ± 40.3%, respectively (Figure 6B). The relative values of Alizarin Red S staining on Day 14 for 0.001, 0.01, 0.1, and 1 μg/mL CMT groups were 120.3% ± 10.7%, 127.4% ± 0.0%, 131.8% ± 23.0%, and 104.6% ± 49.2%, respectively, when the control was considered as 120% (120.2% ± 34.5%) (Figure 6B). 

### 3.5. qPCR Analysis of RUNX2 and COL1A1

**Figure 7:**
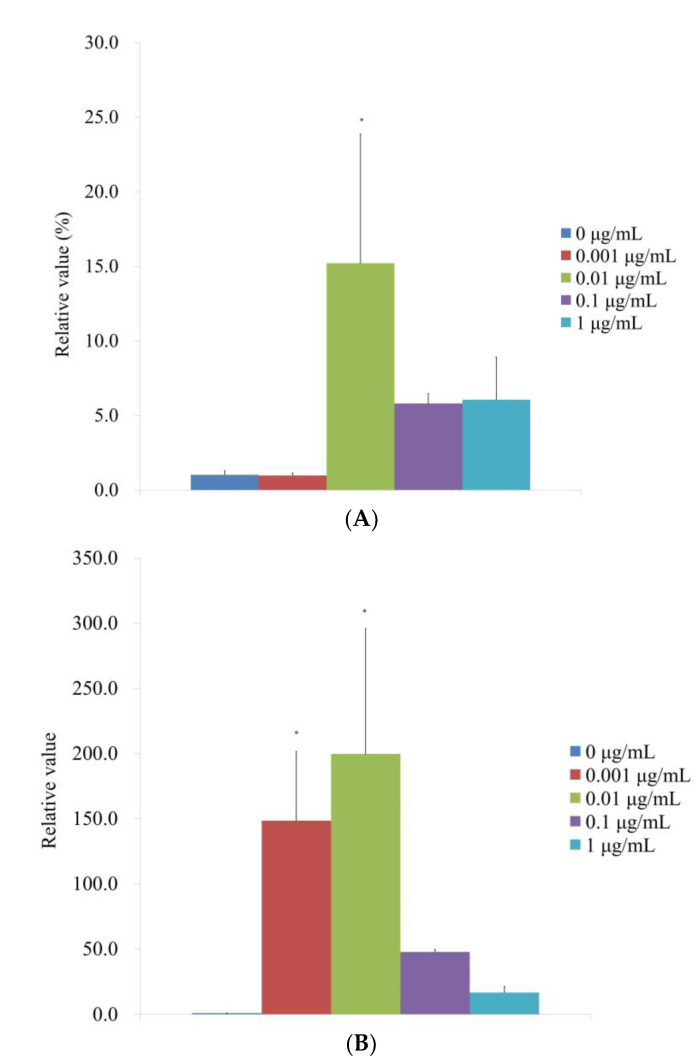
Evaluation of mRNA expression in terms of quantity on Day 14. (**A**) Real-time polymerase chain reaction measurement of RUNX2 mRNA expression on Day 14. * *p <* 0.05 versus the 0 μg/mL on Day 14. (**B**) Real-time polymerase chain reaction measurement of COL1A1 mRNA expression on Day 14. * *p <* 0.05 versus the 0 μg/mL on Day 14.

According to qPCR, the RUNX2 mRNA levels for the 0, 0.001, 0.01, 0.1, and 1 μg/mL CMT groups were correspondingly 1.0 ± 0.3, 1.0 ± 0.2, 15.2 ± 8.7, 5.8 ± 0.7, and 6.1 ± 2.9 on Day 14 (Figure 7A). RUNX2 expression in the 0.01 g/mL group significantly increased with the addition of CMT (*p* < 0.05). qPCR results showed that the mRNA levels of COL1A1 for the 0, 0.001, 0.01, 0.1, and 1 μg/mL were 1.0 ± 0.1, 148.4 ± 52.8, 199.6 ± 96.0, 47.7 ± 1.9, and 16.8 ± 4.6, respectively, for the CMT groups (Figure 7B). At 0.001 and 0.01 g/mL groups, the addition of CMT significantly increased the expression of COL1A1 (*p <* 0.05).

### 3.6. Runt-Related Transcription Factor 2 and Type I Collagen Western Blot Analysis

**Figure 8:**
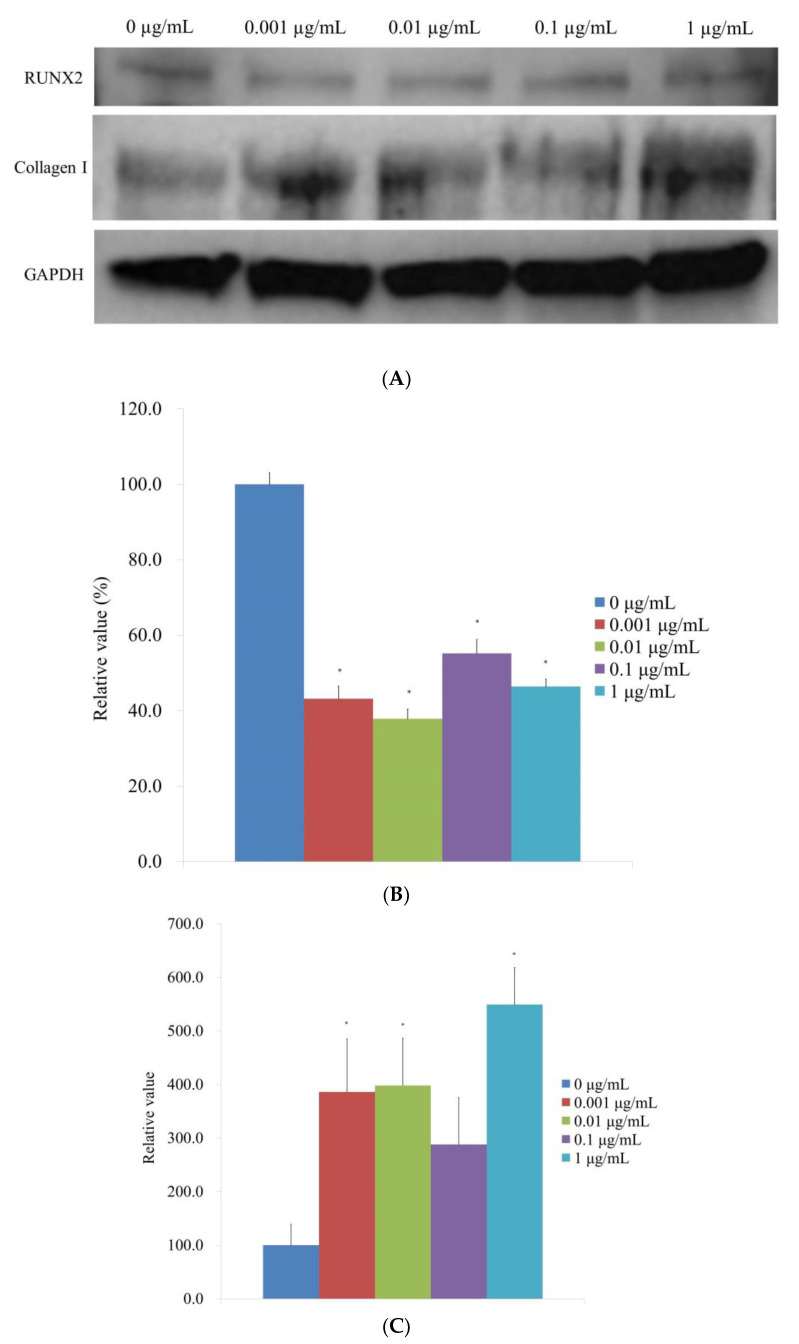
Western blot analysis. (**A**) Type I collagen and Runt-related transcription factor 2 expressions when CMT is added to the culture. (**B**) Normalization of the protein expressions of the Runt-related transcription factor 2. * *p <* 0.05 versus the 0 μg/mL on Day 14. (**C**). Normalization of the protein expressions of the type I collagen. * *p <* 0.05 versus the 0 μg/mL on Day 14.

Following the administration of CMT, a Western blot technique was performed to examine the protein expression of type I collagen and Runt-related transcription factor 2 (Figure 8A). When the protein expressions were normalized, it was discovered that the expression of Runt-related transcription factor 2 was 100.0% ± 3.0%, 43.2% ± 3.3%, 37.9% ± 2.5%, 55.2% ± 3.7%, and 46.4% ± 2.0% for the 0, 0.001, 0.01, 0.1, and 1 μg/mL CMT groups, respectively (Figure 8B). The expression of Runt-related transcription factor 2 was markedly reduced by the addition of CMT (*p <* 0.05). For the 0, 0.001, 0.01, 0.1, and 1 μg/mL CMT groups, normalization of the protein expressions revealed that type I collagen expression was 100.0% ± 39.3%, 386.2% ± 98.9%, 398.1% ± 88.1%, 288.1% ± 87.6%, and 549.2% ± 69.3%, respectively (Figure 8C). At 0.001, 0.01, and 1 g/mL CMT groups, the addition of CMT significantly increased the expression of type I collagen (*p <* 0.05).

## 4. Discussion

In this study, the effects of CMT on human mesenchymal stem cell spheroids’ osteogenic development and mineralization were examined. Alkaline phosphatase activity was used to identify differentiation into an osteogenic lineage, and real-time quantitative PCR and Western blot analysis were used to identify mRNA and protein expression.

Carbonate scaffolds with hydrogel structures offer promising scaffolds for bone tissue engineering applications, regardless of monolayer or spheroid cell culture [23]. In contrast to two-dimensional dedifferentiated fat cells, three-dimensional dedifferentiated fat spheroid promoted osteogenic differentiation and bone formation via canonical Smad 1/5 signaling pathways, according to research comparing the in vitro osteogenic potential of rat dedifferentiated fat cells cultured under osteogenic conditions in three-dimensional spheroids with that in two-dimensional monolayers [24]. The bone regeneration process was accelerated in vivo by neurosphere medium spheroids created under modified neurosphere culture conditions with constant shaking [25]. In contrast, constructions using two-dimensional and three-dimensional bone marrow mesenchymal stem cells behaved comparably in vivo despite a tendency for improved in vitro calcification [23].

RUNX2 is necessary for the growth of osteoblasts and healthy bone production [26]. For the differentiation of osteoblasts and chondrocytes as well as bone production, RUNX2 is a master transcription factor [27]. Numerous osteogenic genes, including alkaline phosphatase and collagen I, are only expressed when RUNX2 is present [28]. This study demonstrated that the use of CMT increased the mRNA expression of RUNX2 and COL1A1, as well as the protein expression of collagen I.

More than 100 secondary metabolites were found in the *Centipeda minima* plant, including terpenoids, flavonoids, mono-phenols, fatty acids, amides, and other types [29]. Sesquiterpene lactones predominate in either the *Centipeda minima* species or many plants of the genus *Centipeda* among them [30]. Sesquiterpene lactones have a variety of biological effects, including immunological responses, hepatoprotective properties, anti-inflammation, anti-cancer, anti-bacteria, anti-allergy, and anti-virus actions [31]. Through the use of the signaling pathways Wnt/-catenin, extracellular signal-regulated kinase, and Jun N-terminal kinase, *Centipeda minima* extract increases the growth of hair and the release of growth factors [11].

It is possible to use combination strategies to improve bone growth. For bone regeneration, a variety of cells was used, including endothelial cell spheroids and uniformly dispersed human adipose stem cells [32]. In a process known as spheroid co-culture, osteocytes and bone-marrow-derived stem cells produce ring-shaped bone-like tissue that improves alveolar bone regeneration [33]. This tissue could be used to encourage bone creation and maturation, speeding up regeneration [33]. Microbiomaterials for bone repair and regeneration were examined using osteoblast-like and human mesenchymal stem cells, and it was discovered that the cells in the spheroids responded to variations in the microbiomaterials’ characteristics, quantities, and the duration of interaction with them [34]. In order to generate a three-dimensional microenvironment for large-defect bone repair, functional spheroids composed of mesenchymal stem cells were tested along with two-dimensional heteronano-layers built of black phosphorus and graphene oxide [35]. Adipose-derived stem cells were used to create composite spheroids, bone-morphogenetic-growth-factor-2-coated nanofibers were used to promote osteogenesis, and the presence of inductive factors modulated the in vitro osteogenic differentiation of adipose-derived stem cells within the biphasic construct while preventing dedifferentiation [36]. 

## 5. Conclusions

In conclusion, CMT had an impact on the osteogenic differentiation of bone marrow-derived stem cell spheroids, and its use may have positive effects on the osteogenic differentiation of cell spheroids.

## Data Availability

This publication contains all of the data that were created or examined during this study.
